# Asymmetric-coupled Ge/SiGe quantum wells for second harmonic generation at 7.1 THz in integrated waveguides: a theoretical study

**DOI:** 10.1515/nanoph-2023-0697

**Published:** 2024-01-18

**Authors:** Enrico Talamas Simola, Michele Ortolani, Luciana Di Gaspare, Giovanni Capellini, Monica De Seta, Michele Virgilio

**Affiliations:** Dipartimento di Scienze, Università degli Studi Roma Tre, Viale G. Marconi 446, 00146, Roma, Italy; Department of Physics, Sapienza University of Rome, Piazzale Aldo Moro 5, I-00185 Rome, Italy; IHP-Leibniz Institut für innovative Mikroelektronik, Im Technologiepark 25, 15236 Frankfurt (Oder), Germany; Dipartimento di Fisica “E. Fermi”, Università di Pisa, Largo Pontecorvo 3, 56127, Pisa, Italy

**Keywords:** second harmonic generation, terahertz, Ge/SiGe quantum wells, non linear waveguide, silicon photonics

## Abstract

We present a theoretical investigation of guided second harmonic generation at THz frequencies in SiGe waveguides embedding n-type Ge/SiGe asymmetric coupled quantum wells to engineer a giant second order nonlinear susceptibility. A characteristic of the chosen material system is the existence of large off-diagonal elements in the *χ* ^2^ tensor, coupling optical modes with different polarization. To account for this effect, we generalize the coupled-mode theory, proposing a theoretical model suitable for concurrently resolving every second harmonic generation interaction among guide-sustained modes, regardless of which *χ* ^2^ tensor elements it originates from. Furthermore, we exploit the presence of off-diagonal *χ* ^2^ elements and the peculiarity of the SiGe material system to develop a simple and novel approach to achieve perfect phase matching without requiring any fabrication process. For a realistic design of the quantum heterostructure we estimate second order nonlinear susceptibility peak values of ∼7 and ∼1.4 × 10^5^ pm/V for diagonal and off diagonal *χ* ^2^ elements, respectively. Embedding such heterostructure in Ge-rich SiGe waveguides of thicknesses of the order of 10–15 μm leads to second harmonic generation efficiencies comprised between 0.2 and 2 %, depending on the choice of device parameters. As a case study, we focus on the technologically relevant frequency of 7.1 THz, yet the results we report may be extended to the whole 5–20 THz range.

## 1 Introduction

The electromagnetic frequency range between 5 and 20 THz contains a rich variety of substance-specific vibrational absorption lines, with countless envisioned applications in astronomy, materials science and biosensing [1], [2]. The lack of efficient and compact sources in this spectral range, however, represents a bottleneck for the further development of technologies such as heterodyne receivers and spectroscopic imaging. As a matter of fact, despite more than two decades of research and with a singular exception achieved leveraging a unique combination of parameters [3], [4], a compact semiconductor laser emitting in the 5–20 THz range has yet to be developed, the strong optical phonon absorption in the Reststrahlen band of III–V compound semiconductors constituting the main roadblock to quantum cascade lasers (QCLs) in this spectral region.

Generation of radiation in the 5–20 THz range is therefore usually obtained via optical rectification in non-centrosymmetric crystals, exploiting the nonlinear optical interaction among the different spectral components of ultrashort VIS and near-IR laser pulses. Although the intrinsic nonlinearity of crystals in the THz range is incredibly high [5], the generation of THz pulses with spectral content above 5 THz from crystals such as ZnTe and GaP is hampered, again, by IR-active phonons. The same holds when leveraging on GaAs and InGaAs based photoconductive antennas [6]. As an alternative, THz generation in gases such as nitrogen has been developed, based on nonlinear optical frequency mixing in the laser-induced plasma of the gas molecules. Broad bandwidths, covering the entire THz gap up to 40 THz, have been demonstrated relying on this approach [7]. To transiently break the molecule symmetry and generate nonlinear effects, however, sophisticated high-peak-power amplified laser systems emitting in the UV–VIS are required [8].

The introduction of materials without IR-active THz phonons, such as electric-gate-biased solid-state films [9], liquids [10], organic polymers [11], and metal-based spintronic THz emitters [12], [13], for nonlinear THz generation has granted improved efficiencies if compared to gas molecule oscillations, mostly due to either the higher dipole density of condensed matter, or to the smaller oscillator mass of electron currents. All these mentioned nonlinear down-conversion approaches still rely on strong laser pulses emitting in the UV, VIS or near-IR to reach measurable levels of THz radiation, and the intrinsic process efficiency remains below 10^−4^ in most cases [8].

High-harmonic generation from low-frequency quasi-continuous wave (CW) optical pumps has also been studied, mostly leveraging the hydrodynamic oscillations of free electrons in 2D as the nonlinear mechanism [14]. Graphene, for example, provides strong intrinsic nonlinear coefficients but has so far required the use of a THz free electron laser pump to obtain measurable signals at high odd harmonics due to its extremely small optical path length, typical of 2D materials [15], [16]. We finally point out that the recent development of novel CW THz gas lasers, in which molecular roto-vibrations are selectively pumped by tunable mid-IR QCLs to obtain population inversion in specific levels, represents a very promising approach to nonlinear frequency manipulations in the THz domain; however only emission frequencies up to 5.5 THz have been demonstrated so far [17], [18].

As an alternative approach, here we investigate the use of highly efficient second harmonic generation (SHG) in waveguides (WG) obtained from epitaxial group-IV semiconductor films, embedding in their active region a stack of n-doped asymmetrically coupled quantum wells (ACQWs). The centro-symmetric character of the diamond lattice implies a vanishing *χ* ^2^ coefficient which suppresses SHG in bulk systems [19]. However, leveraging on the asymmetric character of the ACQW superlattice profile [20], [21], giant *χ* ^2^ values can be artificially engineered to activate SHG. Moreover, the non-polar character of the bulk lattice results in the absence of the Frölich interaction, thus giving access to the Reststrahlen band ubiquitously present in III–V compounds.

The proposed device could be optically pumped with compact semiconductor lasers operating just below the Reststrahlen band (<5 THz), as for instance multi-Watt III–V based QCLs [22], thereby obtaining a relatively compact semiconductor-based THz source covering the 5–10 THz range.

An efficient coupling of a THz QCL source with the frequency-doubling waveguide may be envisioned provided that the thicknesses of their active regions are comparable, so that their fundamental TM modes profiles are well-matched. In this condition, one could exploit a back-to-back alignment achieving an almost physical contact of the two waveguide facets on the two neighboring chips. In such a configuration, a fundamental mode at *ω*/2, excited at the entrance of the WG, transfers through SHG a significant power fraction to the *ω* mode. This physical process has been typically described within the so-called *coupled mode theory*, which allows to evaluate the *ω*/2 − *ω* energy exchange rates as a function of the distance travelled by the pump beam inside the non-linear medium [23].

Leveraging on ACQWs tuned to feature doubly resonant intersubband transitions (ISBT) at *ω*/2, *χ* ^2^ values of the order of 10^5^ pm/V in the mid-IR range have been demonstrated using III–V compound semiconductors [24], [25], [26], [27]. More recently, the non-linear active region has been coupled to a metasurface environment to benefit from a large field enhancement [28], [29], [30]. This approach has been replicated in the SiGe material system, which features lower epitaxy costs compared to compound semiconductors and the overall opportunities of CMOS-compatibility, thus easing mainstream applications of THz nonlinear optics. Focusing on the mid-IR and exploiting ISBTs occurring in valence subbands of p-type Si/SiGe [21] and Ge/SiGe [31] ACQWs, SHG at 25–35 THz with *χ* ^2^ values well above 10^4^ pm/V has been demonstrated.

In this work we theoretically investigate the potential of non-linear SiGe WGs with embedded n-type ACQWs to access via SHG the 5–10 THz region. As a case study, we focus on the *ω* operation frequency of 7.1 THz, which corresponds to both an atmospheric transmission window relevant for astronomy and to a vibrational line of water ice, and that has been proposed to investigate the composition of protoplanetary disks [32], [33]. Frequency-doubling of a III–V QCL emitting at 3.55 THz would then allow one to design a heterodyne receiver at 7.1 THz.

To properly account for the peculiarities of the bandstructure of high-Ge content SiGe alloys which give rise to a non-diagonal *χ* ^2^ tensor, we generalize the coupled-mode theory for guided wave optics [34], removing several approximations ubiquitously present in the literature and appropriate when dealing with simpler semiconductor systems. In the ‘Theoretical Methods’ section we provide an in-depth yet broadly applicable description of our theoretical approach, suitable to simulate guided second harmonic generation regardless of the chosen material system. We then apply the model to a proposed high-Ge content SiGe WG in ‘results and discussion,’ evaluating the effect of various design parameters on SHG efficiency, placing strong emphasis on the features inherent to the distinctive characteristics of the material. In the same section we finally propose a simple yet innovative approach to manage the well-known phase mismatch problem in group IV WGs.

## 2 Theoretical methods

In this work, we theoretically investigate guided SHG leveraging ISBTs of L-point electrons occurring in n-type (001)-Ge/Si_1−*x*_Ge_*x*_ ACQWs. To this aim we investigate structures with doubly resonant transitions among confined subband states. As a first step, the subband eigenstates, their temperature dependent population and the inter-subband oscillator strengths have been estimated by means of a self-consistent multivalley 1D Poisson-Schroedinger solver [35]. This information is then used to calculate the spectral shape of the first *χ* ^1^_*ii*_ and second order optical susceptibility *χ* ^2^_*ijk*_ *i*, *j*, *k* ∈ {*x*, *y*, *z*}, which allowed us to estimate the SHG efficiency *μ* in slab WG devices containing the ACQW stack as active medium. The calculation of *μ* has been performed in the framework of the coupled mode theory, generalizing the approach described in Ref. [23] to account for multi-mode interaction and TE/TM coupling, which in n-type (001)-Ge-rich structures is allowed by the non-diagonal character of the L-point effective mass tensor.

According to coupled mode theory, SHG depends on the overlap integrals among propagating modes sustained by the WG at *ω* and *ω*/2. To obtain these terms, we solve the Maxwell equation for both TE and TM modes assuming lossless media. This problem admits an analytical solution when the substrate, WG, and cover regions are each constituted by a homogeneous medium. However, Ge-on-Si epitaxy requires the introduction of a virtual substrate (VS) layer between the silicon substrate and the Ge-rich ACQW-region to accommodate the lattice mismatch between Si and Ge [36], as detailed in the following section. Therefore we relied on a numerical treatment to properly account for this additional layer.

For simplicity we assume a slab-type WG geometry. We set a reference frame where light propagates along *z* and the *x* axis is chosen parallel to the growth direction, implying translation invariance in the (*yz*) plane. It follows that the field amplitude **E** can be written as **E**(*x*) = *AE*(*x*) where, following Ref. [34] the normalization constant *A* is chosen in such a way that each mode carries a power flow of |*A*|^2^ W per unit length along the *y* direction. To calculate the refractive index spatial profile, the permittivity tensor *ε*_*jj*_ with *i* ∈ {*x*, *y*, *z*} in the ACQW active region has been estimated using the effective medium approach described in Ref. [37]. Thus, considering the individual contributions of barrier and well layers we get

(1) $$\varepsilon_{jj}=\left(1-f\right)\varepsilon_{b}+f\varepsilon_{w}+Re\left\{\chi_{jj}^{1}\right\}\;\;\;\text{for}\;\;j\;=\;y,z$$

(2) $$\varepsilon_{xx}={\left(\frac{1-f}{\varepsilon_{b}}+\frac{f}{\varepsilon_{w}}-\frac{R\left\{\chi_{xx}^{1}\right\}}{\varepsilon_{w}^{2}}\right)}^{-1}$$

where *ε*_*w*_ and *ε*_*b*_ are the relative permittivities of well and barrier, respectively, and *f* is the ratio between the cumulative thickness of the two QWs and that of the complete ACQW module. Notice that in the above equations we have also taken into account the contribution to *ε*_*jj*_ due to the real part of the first order optical susceptibility tensor $Re\left\{\chi_{jj}^{1}\right\}$ associated to the ACQWs and calculated at 77 K. Finally, *ε*_*b*_ in the Si_1−*x*_Ge_*x*_ barrier layer is obtained by linear interpolation of the Si and Ge permittivity values.

We approach the problem of guided SHG according to the coupled-mode formalism [38], expanding upon the existing literature to tailor the modelling of SHG to the peculiarities of the proposed material system. Specifically, we drop the assumption of having only two interacting modes, ubiquitously present in literature, considering instead the coupling among all the WG sustained modes. This generalization allows us to calculate the dynamical evolution along the propagation direction of a generic modal power distribution defined at the WG entrance *z* = 0. Moreover, tracking concurrently all mode interactions becomes essential for Ge-rich quantum structures due to the presence of significant off-diagonal elements in the *χ* ^2^_*ijk*_ tensor, associated to dipoles with a non-vanishing component in the growth plane [39], [40]. Indeed, in parallel to SHG processes originating from interactions among TM modes only, off-diagonal terms like *χ* ^2^_*yyx*_ and *χ* ^2^_*yxy*_ couple modes with different polarizations, allowing SHG processes involving two TE and one TM mode. On the other hand, we find *χ* ^2^_*ijk*_ elements with an even number of ‘*x*’ indexes to be zero, as expected from symmetry considerations.

In this framework the spatial evolution of the *z* dependent modal amplitude *A* is obtained solving the system of coupled first-order differential equations given by

$$\left\{\begin{array}{c} \frac{dA_{m,u}}{dz}=\frac{-\alpha_{m,u}}{2}A_{m,u}-\frac{i\omega \varepsilon_{0}}{4}\underset{n\in \left\{\frac{\omega }{2}\right\}}{\sum }\underset{p\in \left\{\frac{\omega }{2}\right\}}{\sum }\underset{v}{\sum }\underset{w}{\sum }A_{n,v}\times A_{p,w}\times \chi_{u,v,w}^{2}\times S\left(\ldots \right)\times e^{-i\left(\beta_{n}+\beta_{p}-\beta_{m}\right)z}\\ \;\forall m\in \left\{\omega \right\};u,v,w\in \left\{x,y,z\right\}\;\left(3a\right)\\ \frac{dA_{n,v}}{dz}=\frac{-\alpha_{n,v}}{8}A_{n,v}-\frac{i\omega \varepsilon_{0}}{4}\underset{m\in \left\{\omega \right\}}{\sum }\underset{p\in \left\{\frac{\omega }{2}\right\}}{\sum }\underset{u}{\sum }\underset{w}{\sum }A_{m,v}\times A_{p,w}^{*}\times \chi_{u,v,w}^{2}\times S\left(\ldots \right)\times e^{-i\left(\beta_{m}-\beta_{n}-\beta_{p}\right)z}\\ \;\forall n\in \left\{\frac{\omega }{2}\right\};u,v,w\in \left\{x,y,z\right\}\;\left(3b\right) \end{array}\right.$$

(4) $$S\left(m,n,p,u,v,w\right)=\int^{\mathrm{active}}E_{m,u}\left(x\right)E_{n,v}\left(x\right)E_{p,w}\left(x\right)dx$$

where *A*_*m*,*u*_ is the amplitude of mode *m* along reference axis *u*, *S* is the modal overlap integral to be performed in the active region featuring a non-vanishing $\chi_{u,v,w}^{2}$ , *ω* is the frequency of the second harmonic, {*ω*} and {*ω*/2} indicate the sets of guide-sustained modes at frequencies *ω* and *ω*/2, respectively, over which the summations must be performed; finally *b*_*m*_ is the propagation wavevector associated to mode *m*, obtained solving the Maxwell equation. The system includes an equation of the form of 3a for each mode in {*ω*} and one of the form of 3b for each in {*ω*/2}. Each equation features an absorption term and a sum of coupling terms, describing the power exchange among the guide sustained modes.

Regarding the absorption term, it “removes” a-posteriori the assumption of a lossless medium [41], the absorption coefficients *α*_*m,u*_ for mode *m* and axis index *u* obeying the following equation

(5) $$\alpha_{m,u}=\frac{\omega^{2}}{\beta_{m}c^{2}}\frac{\int Im\left\{\chi_{uu}^{1}\left(x\right)\right\}E_{m,u}^{2}\left(x\right)}{\int E_{m,u}^{2}\left(x\right)}$$

where *χ* ^1^ is a multi-step function of *x* to represent the stack of different layers (the dependence on *ω* left implied), each with its own electric permittivity, resulting from the joint contribution of ISBTs in the active region and free carrier absorption in the other layers. Specifically, we evaluated the latter in the Si substrate for a background doping concentration of 5 × 10^14^ cm^−3^ following Ref. [42].

Under the specific approximation of a slab-type WG, the electric field of TE modes is parallel to *y*; in the general case (TM mode and/or ridge WG), however, the orientation of the electric field for both TE and TM modes is slightly tilted with respect to the cartesian axis of reference (*y* and *x*, respectively). We therefore let the indexes *u*, *v* and *w* run over the entire {*x*, *y*, *z*} cartesian set. The resulting power in mode *m* is thus given by the square modulus of the vectorial amplitude ***A***_*m*_.

Each coupling term results from the product of the field amplitudes of the involved modes with the corresponding *χ* ^2^ element, the modal overlap integral and an exponential term. The latter oscillates along the propagation direction with a spatial frequency equal to Δ*β* = *β*_*m*_ − *β*_*n*_ − *β*_*p*_ [27]. It follows that if the difference of the propagation vectors is nonzero the system manifests a back-and-forth modulation in the power transfer between the pump mode at *ω*/2 and the second harmonic mode at *ω*, with a characteristic dephase length *L*_*φ*_ = π/Δ*β*. The main limitations to high SHG efficiency are thus the existence of a phase mismatch cumulated at the WG exit facet and the absorption depleting the pump signal with a characteristic length of *L*_*α*_ = 1/*α*. A comparison between these two spatial quantities indicates which of the two effects limits the SHG efficiency for any given configuration.

## 3 Results and discussion

**Figure 1:**
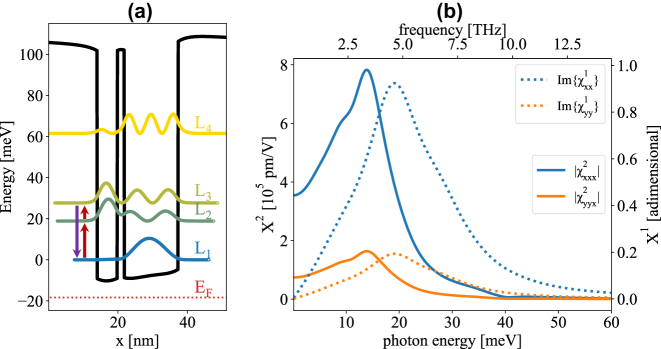
Optical susceptibility of the active region. (a) L-point band-edge profile and subband eigenstates of the investigated asymmetric coupled quantum well structure Ge/Si_18_Ge_82_/Ge/Si_18_Ge_82_ (thickness 5.8/2/15.5/12 nm) calculated at 77 K. Dopant concentration in the wells and the tunneling barrier is set to 1 × 10^17^ cm^−3^. (b) Diagonal (*xxx*) and off-diagonal (*yyx*) spectral shape of the second order non-linear susceptibility tensor (solid). Imaginary part of the linear optical susceptibility for TM (*xx*) and TE (*yy*) polarization is also shown (dotted).

The active region comprises a periodic repetition of n-type ACQW modules featuring a pair of Ge QWs with thicknesses of 5.8 and 15.5 nm, coupled by a 2 nm Si_18_Ge_82_ tunneling barrier. Adjacent ACQWs are separated by a 12 nm intrinsic Si_18_Ge_82_ spacer, thick enough that their coupling is negligible. Given the recent developments in SiGe CVD epitaxy [43], [44], such proposed design is challenging but realistic. Figure 1a shows the L-band edge profile and the confined eigenstates, marked as *L*_*n*_. Eigenstates *L*_1_, *L*_2_, and *L*_3_, are the ones involved in the doubly resonant transitions. Figure 1b reports the resulting diagonal and off-diagonal spectral shape of the *χ* ^2^ tensor (solid) and of the imaginary part of the linear susceptibility Im{*χ* ^1^} (dotted), calculated at 77 K, assuming a 10^17^ cm^−3^ n-type doping concentration in the entire ACQW structure, corresponding to an N_2D_ equal to 2 × 10^11^ cm^−2^. Results reported henceforth refer to this fixed combination of doping and temperature; some exploratory simulations obtained varying these two parameters are discussed in the Supplementary.

The ratio between the modulus of off-diagonal and diagonal *χ* ^2^ components and the ratio between Im{*χ* ^1^_*zz*_} and Im{*χ* ^1^_*xx*_} are both equal to the ratio between the TE and TM oscillator strengths. The latter is given by (*m*_*T*_ − *m*_*L*_)^2^/(2*m*_*L*_ + *m*_*T*_)^2^, representing the square ratio between the *zz* and *xx* elements of the inverse effective mass tensor, whose principal axis is along the [111] crystallographic direction [45]. With a Ge longitudinal effective mass *m*_*L*_ of 1.59 *m*_0_ and a transverse effective mass *m*_*T*_ of 0.093 *m*_0_ [46] such ratio is equal to 0.21. This non-negligible value supports the necessity of including the off-diagonal coupling terms in the numerical modeling of optical non-linearities in WGs based on this material system.

Since most of the carriers populate *L*_1_, the position of the *χ* ^2^ resonance roughly corresponds to (*L*_3_ − *L*_1_)/2, while Im{*χ* ^1^} peaks at *L*_2_ − *L*_1_. Equally spaced levels would therefore result in *χ* ^2^ and Im{*χ* ^1^} featuring their maxima at the same energy. Detuning the double resonance [47], i.e. shifting *L*_2_ towards *L*_3_ while keeping the distance between *L*_1_ and *L*_3_ constant, blueshifts the Im{*χ* ^1^} maximum, therefore reducing pump absorption. Starting from a perfectly double resonant design with coupled QWs of 7.0 and 14.5 nm, we tailor their thicknesses to obtain the most effective energy detuning. We find the optimal values to be 5.8 and 15.5 nm, placing the *L*_1_ → *L*_3_ transition at roughly 28 meV and the *L*_1_ → *L*_2_ one slightly below 19 meV. We stress that the adopted value of the FWHM of the ISBT, for which we assume a Lorentian broadening, is an important parameter for the evaluation of the SHG efficiency. A lower FWHM results in sharper resonant features and higher maximum values for *χ* ^2^ and Im{*χ* ^1^}, impacting the overlap of the *χ* ^2^ and Im{*χ* ^1^} spectra in detuned systems, reducing absorption at *ω*/2. In our calculations, every optical transition has been assigned the same FWHM, corresponding to the value of 6.5 meV extrapolated from FTIR measurements performed on similar samples [48].

At 77 K we find that the *χ* ^2^_*xxx*_ element reaches the impressive peak value of 7.7 × 10^5^ pm/V. This number is of the same magnitude as those obtained for comparable ISBT architectures featured by non-linear WGs operating in other spectral windows (pioneering [49], [50] but also, more recently [51], [52]). Notice that in Ge-rich devices the relatively small value of the out-of-plane confinement effective mass (0.13 *m*_0_) in the *L* subband favors high *χ* ^2^ values. In parallel, for a given carrier density, the 4-fold degeneracy of the L-point band edge, in conjunction with the relatively high value of the in-plane effective mass (0.32 *m*_0_), results in a large density of states, lowering the Fermi energy. This effect reduces the thermal quenching of the *L*_1_ → *L*_2_ transition, thus improving the temperature robustness of the system.

**Figure 2:**
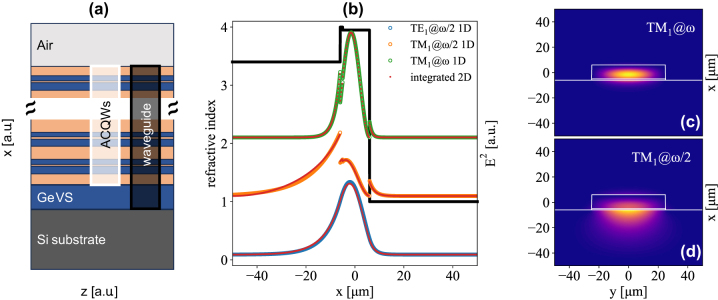
Optical modes supported by the waveguide. (a) Multi-layer material stack of the non-linear waveguide, highlighting the ACQWs embedded in the guiding region. (b) Refractive index profile of the slab WG along the growth direction. Normalized profiles of the square modulus of the electric field for the TE_1_ and TM_1_ modes @*ω*/2 (blue and orange, respectively) and the TM_1_@*ω* (green) are also shown. The red curves represent the corresponding profiles obtained integrating along y the modes of a 50 µm wide ridge-WG, shown for TM_1_@*ω* and TM_1_@*ω*/2 in (c) and (d), respectively. Pump frequency is set at 3.55 THz.

Within the constraints given by the material system, we now design a slab-type WG suitable for guided SHG. A prime advantage of Ge-rich alloys is their possibility to be epitaxially grown on a standard silicon substrate which, featuring a smaller refractive index than germanium (3.4 and 4.0, respectively) directly acts as the bottom cladding layer of the WG, air being the top one. Such growth requires the introduction of VS between the silicon substrate and the active region to reduce the threading dislocation density (TDD) induced by the Si/Ge lattice mismatch. Among the different strategies pursued in the last years, the current consensus suggests the best results are achieved using a thick germanium VS layer in conjunction with a reverse graded buffer i.e. a stack of SiGe layers with decreasing Ge content [53], [54]. Given the extremely high average germanium concentration of the active region comprising the ACQWs (93 %) it is reasonable to assume the reverse grading part to be of negligible thickness when compared to the germanium VS and the ACQW region. We therefore omit these graded layers in the calculations. The WG itself is thus constituted by the combination of a germanium VS and the ACQW region (see Figure 2a), modeled using equations (1) and (2). To have confined modes for wavelengths around 85 μm (3.55 THz), given the refractive index of ∼4, it is necessary to reach a total WG thickness of about 10 μm. It has been shown that increasing the thickness of the VS leads to a significant reduction of the TDD thus improving the quality of the material [55]. However, for a fixed total thickness of the WG, such an increase would hamper SHG by reducing the fraction of optical power in the ACQW region. In the following the thickness of the germanium VS is therefore fixed at 1 μm since this value is sufficient to reduce the TDD to ∼10^7^ cm^−2^.

We assume the width of the WG to be much larger than its total thickness, allowing us to treat the problem using a 1D slab-type geometry, which is computationally much faster than a 2D one. For a thickness of 10 μm at both *ω* and *ω*/2 the WG allows a single TE and TM mode. Upon decreasing the thickness, TM_1_@*ω*/2 disappears below 9.4 μm while for larger thicknesses higher order modes start to be supported; specifically, TE_2_@*ω* and TM_2_@*ω* appear at approximately 14.1 and 15.8 μm, respectively. Figure 2b shows the normalized profiles of the square moduli of the electric field for all the modes supported by a 12 μm thick WG except TE_1_@*ω* which is not involved in SHG. In the figure we also plot the refractive index profile seen by TM_1_@*ω* (modes at different energy and/or polarization experience slightly different refractive indexes in the active region as detailed in the theoretical section). We have numerically verified that for ridge WGs of thickness ∼10 μm featuring lateral width ≥50 μm, the 2D modes can be treated with a 1D approach. Figure 2c and d report the squared electric fields for modes TM_1_@*ω* and TM_1_@*ω*/2, respectively, calculated in a 12 μm thick and 50 μm wide ridge waveguide. Integrating a squared electric field distribution along the *y* axis yields a mode profile which is practically indistinguishable from the one obtained in the slab configuration, as shown in Figure 2b. Besides the electric field distribution, solving the Maxwell equation yields the propagation wave-vector *β* for each mode, allowing us to set an arbitrary power distribution among the modes at the WG entrance (*z* = 0) and calculate its evolution along the propagation axis by integrating Eqs. (3).

Since the entrance facet in ridge WGs has a finite area, SHG efficiencies are traditionally given in units of [% W^−1^], under the weak signal assumption which neglects any saturation effects. Switching to a slab WG, which is invariant along *y*, requires a normalization of the efficiency along such direction. *μ* is then measured in units of [% μm W^−1^].

Since our model removes the weak signal assumption and fully considers saturation effects, *μ* must be referred to a specific ‘probe’ input power (in units of [W/μm]), thus yielding efficiencies in units of [%]. Specifically, throughout this paper, we set a constant input power of 10 mW/μm in either TM_1_@*ω*/2 or TE_1_@*ω*/2; SHG in TM@*ω* is thus activated by *χ* ^2^_*xxx*_ and *χ* ^2^_*yyx*_, respectively. We calculate the *z*-dependent efficiency *μ*_m_ for *ω* photons in mode *m* as ***|A***_*m*_ *(ω,z*)|^2^/| ***A*** (*ω*/2,0)|^2^. For a ridge WG 100 μm wide, a conversion factor of 1 is to be adopted to compare the SHG efficiencies here reported as [%] with the traditional ones expressed in [% W^−1^].

**Figure 3:**
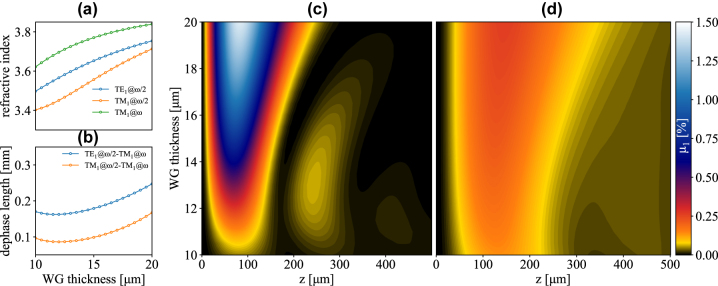
SHG efficiency under TE and TM pumping. (a) Effective refractive indexes of the TM_1_ modes @*ω*/2 and @*ω* and of the TE_1_ mode @*ω*/2, as functions of the WG thickness. (b) Dephase lengths L_φ_ for TM_1_@*ω*/2-TM_1_@*ω* and TE_1_@*ω*/2-TM_1_@*ω* SHG. (c) TM_1_@*ω*/2-TM_1_@*ω* and d) TE_1_@*ω*/2-TM_1_@*ω* SHG efficiency as a function of waveguide thickness and propagation length. Pump frequency is set at 3.55 THz in all plots.

As apparent from Figure 2, *ω*/2 modes are weakly confined, featuring significant leakage in the Si substrate. On the other hand, *ω* modes are more tightly confined in the Ge-rich region, thus experiencing larger effective refractive indexes. This mismatch results in relatively short dephase lengths (<100 μm). It follows that increasing the thickness of the WG improves the confinement of the *ω*/2 modes, leading to smaller refractive index mismatches (Figure 3a) and larger dephase lengths *L*_φ_ (Figure 3b). It is worth noticing that a divergence in the dephase length between TM_1_@*ω* and TM_1_@*ω*/2 can be in principle obtained for a well-defined value of the WG thickness. Recalling that the refractive index depends on Re{*χ* ^1^} and that Re{*χ* ^1^(*ω*/2)}>Re{*χ* ^1^(*ω*)} since Im{*χ* ^1^} peaks at *ω*/2, it follows that there must exist a given value of the total WG thickness where the confinement of TM_1_@*ω*/2 becomes strong enough that its effective refractive index perfectly matches that of TM_1_@*ω*. According to our calculations this occurs for a thickness of approximately 32.5 μm, unfortunately a value currently unreachable by state-of-the-art epitaxy. As a matter of fact, although we have numerically investigated WG thicknesses up to 20 μm, the largest experimentally accessible values are currently around 15 μm [43], [56].

A large *L*_*φ*_ boosts *μ*_1_ as shown in Figure 3c (3d) where we plot the SHG efficiency associated to TM_1_@*ω* as a function of propagation length and WG total thickness** **upon pumping TM_1_@*ω*/2 (TE_1_@*ω*/2). For dephase lengths shorter than *L*_*α*_ the optical power distribution between the pump mode and the second harmonic mode TM_1_@*ω* oscillates along *z*. This regime is clearly distinguishable in the bottom part of Figure 3c only, as in the top part, where *L*_*φ*_ surpasses *L*_*α*_, *μ*_1_ becomes capped by the absorbance of the active region and oscillations are suppressed. A similar oscillatory behavior is also observed in Figure 3d upon pumping the TE_1_@*ω*/2 mode, although the oscillations are now partially quenched by the large difference between the absorption coefficients of TE_1_@*ω*/2 and TM_1_@*ω* photons.

From a quantitative point of view, for a WG thickness of 15 μm the estimated SHG efficiency featured by a TM_1_@*ω*/2 (TE_1_@*ω*/2) pump reaches the value of 0.79 % (0.22 %), peaking after a propagation length of 71 μm (126 μm). Notice that the relative magnitude of the two efficiencies (x4) is significantly lower than (*χ* ^2^_*xxx*_/*χ* ^2^_*yyx*_)^2^ ∼ 1/0.21^2^ ∼ 4.8^2^, the factor one would expect focusing exclusively on the *χ* ^2^ tensorial properties previously discussed. This discrepancy highlights the relevant role of the different absorption coefficients and dephase lengths associated to TE and TM photons. In particular, *L*_*α*_ is significatively larger in TE since Im{*χ* ^1^_*xx*_} and Im{*χ* ^1^_*yy*_} are in the same ratio of 4.8. As *L*_*φ*_ is also larger, it follows that the SHG process under TE_1_@*ω*/2 pumping benefits from a longer propagation length, thus partially compensating for the smaller *χ* ^2^ element.

**Figure 4:**
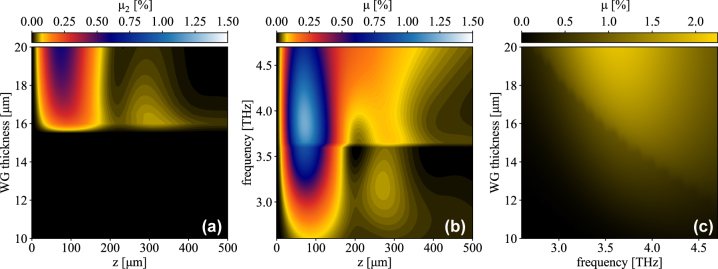
Mode and frequency resolved SHG efficiency. (a) TM_2_@*ω* SHG efficiency as a function of waveguide thickness and propagation length for a pump frequency of 3.55 THz (TM_2_@*ω* is supported only for thicknesses > 15.8 µm). (b) ∑_*n*_TM_*n*_@*ω* total SHG efficiency as a function of pump frequency and propagation length for a WG thickness of 15 μm. (TM_2_@*ω* is supported only for frequencies >3.65 THz). (c) Peak value along the propagation direction of the ∑_*n*_TM_*n*_@*ω* total SHG efficiency. In all plots the TM_1_@*ω*/2 mode is pumped at the WG entrance.

As previously mentioned, TM_2_@*ω* starts to be supported for WG thicknesses above 15.8 μm. Broadly speaking, its associated SHG efficiency *μ*_2_ is significantly lower than that of TM_1_@*ω* (*μ*_1_) due to tinier modal overlap integrals *S* (the mode profile of TM_2_@*ω* features a node in correspondence of TM_1_@*ω*/2’s and TE_1_@*ω*/2’s maxima). Yet it would be reckless to neglect TM_2_@*ω* in the calculations, as it experiences a different effective refractive index with respect to TM_1_@*ω* and may therefore benefit from more favorable matching conditions. Figure 4a reports SHG efficiency in TM_2_@*ω* *μ*_2_ with a TM_1_@*ω*/2 pump; considering a TE_1_@*ω*/2 pump instead, TM_2_@*ω* suffers from very unfavorable mismatch conditions and the related efficiency is found to be negligible. With respect to the TE@*ω* modes, their excitation requires the joint pumping of TM_1_@*ω*/2 and TE_1_@*ω*/2 due to the *χ* ^2^ symmetry properties previously discussed, a case we have not investigated thoroughly.

Fixing the WG thickness to 15 μm, Figure 4b reports, as a function of pump frequency and propagation length, the combined SHG efficiency *μ* obtained summing *μ*_1_ and *μ*_2_, when exciting TM_1_@*ω*/2. Remarkably, although the proposed ACQW design targets a 3.55 THz pump, SHG is non-negligible for a large bandwidth around this frequency. Such large bandwidth stems from the relatively large FWHM of the active ISBTs, which however limits the peak *χ* ^2^ value. It also highlights a discontinuity at approximately 3.7 THz stemming from the appearance of TM_2_@*ω*. This enhances the overall SHG efficiency, as apparent from the figure. The peak efficiency is obtained for a 3.8 THz pump since increasing the photon frequency results in a tighter mode confinement, overcompensating for the off resonance *χ* ^2^ value. Figure 4c shows the maximum SHG efficiency along *z* as a function of *ω*/2 and WG thickness. In this case the appearance of TM_2_@*ω* creates a discontinuity along the top-left/bottom-right diagonal which separates the single-mode WG regime from the multi-mode one. Similar results are found when pumping TE_1_@*ω*/2.

So far, we have restricted this theoretical investigation to slab-type WGs employing only silicon, germanium and their alloys, avoiding more complex solutions such as grated WGs to suppress phase mismatch [57] and double-surface plasmon structures to improve TM-modes confinement [58]. We followed this guideline to avoid over-bloating the scope of the paper, which is to evaluate the feasibility of SHG in the THz rather than to determine an optimized design. To evoke an idea of the possibilities, we will nonetheless conclude by reporting a rather simple yet innovative approach to refractive index engineering.

**Figure 5:**
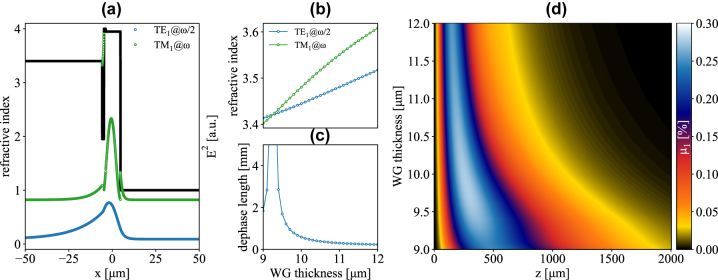
Enhanced SHG efficiency using a GeOI substrate. (a) Refractive index profile and normalized square moduli of the electric field for the TE_1_@*ω*/2 and TM_1_@*ω* modes when using a GeOI substrate. (b) Effective refractive indexes of the TE_1_@*ω*/2 and TM_1_@*ω* modes as functions of the WG thickness. (c) Dephase length for TE_1_@*ω*/2- TM_1_@*ω* SHG as a function of the WG thickness. (d) TE_1_@*ω*/2- TM_1_@*ω* SHG efficiency as a function of waveguide thickness and propagation length on an GeOI substrate.

Employing the same exact ACQW and a similar WG configuration, we now assume the epitaxy to be performed on a GeOI substrate instead of a standard Si one, the only significant difference being the presence of a silicon dioxide layer in between the Si substrate and the 1 μm thick Ge VS layer. In the following, we set the SiO_2_ thickness at 1 μm and rely on Ref. [59] to model its optical properties, tweaking equation (5) accordingly. The adoption of a GeOI substrate has a negligible impact on TE modes, while it causes radical variations in the TM ones because of their boundary conditions. Specifically, no TM modes @*ω*/2 are supported by the WG up to thicknesses of approximately 14 μm, while TM modes @*ω* experience smaller effective refractive index, which can be exploited to tune the phase mismatch in TE/TM SHG. Figure 5a reports the normalized profiles of the square moduli of the electric field for TE_1_@*ω*/2 and TM_1_@*ω*, for a total WG thickness of 9.5 μm. Effective refractive indexes are shown in Figure 5b as a function of WG thickness, evidencing a crossing point at approximately 9.25 μm, which corresponds to a perfect phase match as indicated by the divergence of the dephase length plotted in Figure 5c. Thus, at this particular WG thickness, the only factor limiting SHG is the absorption from ISBTs in the active region and free carriers in the substrate, allowing to reach efficiencies >0.2 % as shown in Figure 5d. Notice that using Si substrates, similar* μ *values could be achieved only for WG thickness above 15 μm (see Figure 3d).

## 4 Conclusions

We have calculated the efficiencies of guided SHG processes in the THz spectral range for non-linear Ge-rich SiGe WGs with embedded ACQWs. As an exemplificative case we have targeted the scientifically relevant frequency of 7.1 THz, currently unreachable by conventional laser sources, finding *χ* ^2^ values at the 10^5^ pm/V scale, resulting in efficiencies comprised between 0.2 and 2 % depending on the choice of design parameters, for interaction lengths of the order of 100 μm. The studied material system provides the clear advantages of lower costs and CMOS compatibility over its III–V counterpart. Yet it also features a conduction band structure resulting in a *χ* ^2^ tensor with significant, non-vanishing off-diagonal elements. The proper treatment of the guided SHG has thus required the development of a numerical model capable of concurrently tracking every *χ* ^2^-mediated interaction among all the supported modes. Equipped with this model, we have methodically discussed the peculiarities of SHG in non-linear SiGe WGs and proposed a simple material-specific approach which leverages on such peculiarities to boost the SHG efficiency by effectively solving the phase mismatch problem.

Although we picked the second harmonic frequency of 7.1 THz and tailored the ACQW design accordingly, we point out that the entire 5–20 THz spectral range is accessible by the chosen material system, the upward limit set by the conduction band offset between wells and barriers of ∼130 meV.

Furthermore, we point out that, since SHG is a special case of the more general mechanism of sum frequency generation, the n-type Ge/SiGe ACQW architecture here investigated can be tailored towards this application as well. The results of our calculations, combined with the recent advancements in group-IV epitaxy, represent a first step towards the experimental demonstration of SHG in the THz using Ge/SiGe ACQWs.

## Supplementary Material

Supplementary Material Details
