# Supplementary material for: Asymmetric-coupled Ge/SiGe quantum wells for second harmonic generation at 7.1 THz in integrated waveguides: a theoretical study
Source: Nanophotonics. 2024 Jan 18;13(10):1781–91. doi: 10.1515/nanoph-2023-0697 (PMC11501687; doi:10.1515/nanoph-2023-0697)
Supplement: Supplementary file 1 — Supplementary Material Details [file j_nanoph-2023-0697_suppl_001.docx]

**Supplementary Information**

**Asymmetric-coupled Ge/SiGe quantum wells for second harmonic generation at 7.1 THz in integrated waveguides: a theoretical study**

Enrico Talamas Simola^1^, Michele Ortolani^2^, Luciana di Gaspare^1^, Giovanni Capellini^1,3^, Monica De Seta^1^, and Michele Virgilio^4^

1. Dipartimento di Scienze, Università degli Studi Roma Tre, Viale G. Marconi 446, 00146, Roma, Italy
2. Department of Physics, Sapienza University of Rome, Piazzale Aldo Moro 5, I-00185 Rome, Italy
3. IHP-Leibniz Institut für innovative Mikroelektronik, Im Technologiepark 25, 15236, Frankfurt (Oder), Germany
4. Dipartimento di Fisica “E. Fermi”, Università di Pisa, Largo Pontecorvo 3, 56127, Pisa, Italy

Given the exploratory nature of this work, we decided to constrain the optimization of the proposed device to a limited set of device parameters. In this supplementary information, we provide insight regarding the effects one would have by changing some of those parameters which were kept constant throughout the main paper. Therefore, unless specifically stated, all parameters in the calculations reported here mirror those of the main text.

**Considerations on doping**

Increasing the doping concentration density in the ACQW structure boosts the magnitudes of both χ^1^ and χ^2^. Qualitatively speaking, the apparent advantage of having a larger χ^2^ is therefore somewhat blunted by the larger absorption (Im{χ^1^}).

To shed light on the interplay between these two opposite effects, in Figure S1a we show the results of SHG efficiency calculations performed at the ω/2 frequency of 3.55 THz with a fixed WG thickness of 15 μm using an ACQW structure identical to the one shown in Figure 1a of the main text, the only difference being the doping concentration which is let to vary a factor of 4 above and below the value of 10^17^ cm^-3^ used in the main text.


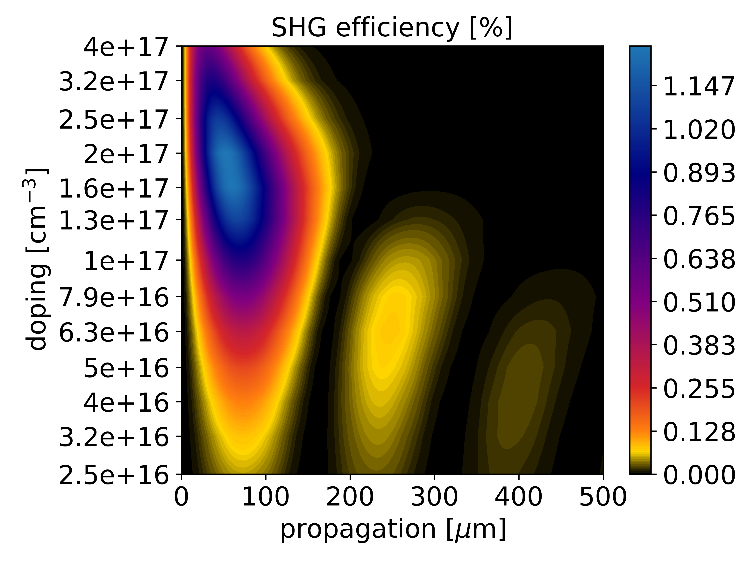

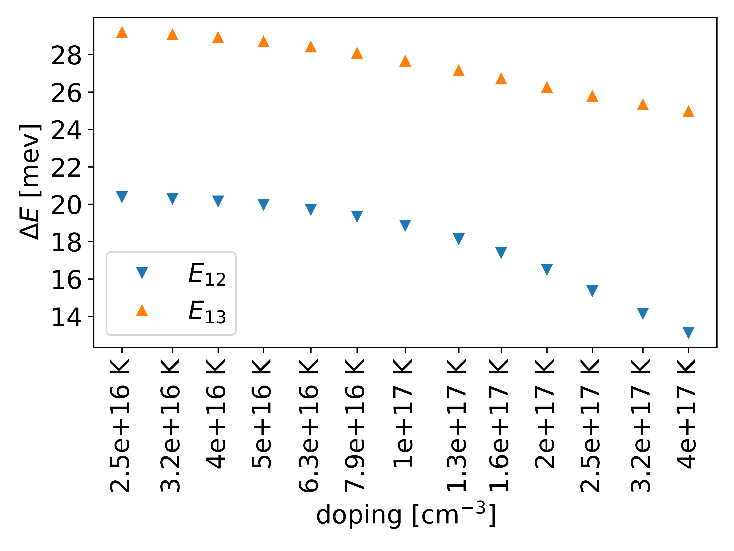


Figure S1: a) SHG efficiency as a function of doping and propagation length. WG thickness is 15 μm, ω/2 = 3.55 THz. b) Energies of the intersubband transitions involved in SHG as a function of doping density.

As doping concentration decreases below 10^17^ cm^-3^ the peak SHG efficiency crumbles. This is mainly a consequence of the phase mismatch overtaking the pump absorption as the main limiting factor to SHG. Such mechanism is accompanied by the appearance of oscillations in the SHG efficiency along the propagation direction, for which hold considerations analogous to those reported in the main text when discussing the effects of WG thickness and commenting Figure 3c.

As doping increases above 10^17^ cm^-3^, the peak SHG efficiency increases up to 2x10^17^ cm^-3^ then for higher doping concentration drops once again. This curious behavior may is to be attributed to the effect of doping on the subband energies: a variation in the doping concentration may significantly shift the spacing of the intersubband levels, resulting in an ACQW structure which is no longer optimized to achieve the highest SHG efficiency. Figure S1b reports the energies of the intersubband transitions involved in SHG as a function of doping density, demonstrating how higher doping leads to a redshift of both E_12_ and E_13_. The former is particularly affected, detrimentally impacting the detuning which had been optimized for a concentration of 1x10^17^ cm^-3^.

It follows that an ACQW structure with optimal detuning may be designed only once doping concentration is known: a proper reassessment of the data shown in Figure S1a would in principle require an optimization of the detuning for each doping level. While this is clearly beyond the scope of this document, as an exemplary case we did indeed perform such optimization for the 4x10^17^ cm^-3^ doping density.

Figure S2 compares two ACQW designs with a doping concentration of 4x10^17^ cm^-3^. The one in S2a has the same QWs thicknesses of 5.8 and 15.5 nm as in the main text, while the one in S2b features optimized thicknesses of 5.0 and 15.0 nm, respectively. The variation is rather small, nevertheless sufficient to push E_12_ above E_13_/2, increasing detuning and SHG efficiency μ which now peaks at 1.4%, as shown in Figure S2c.


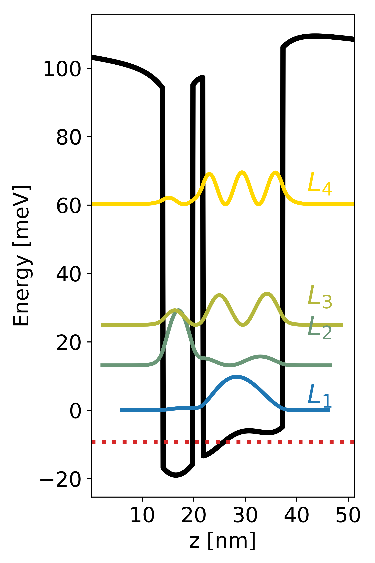

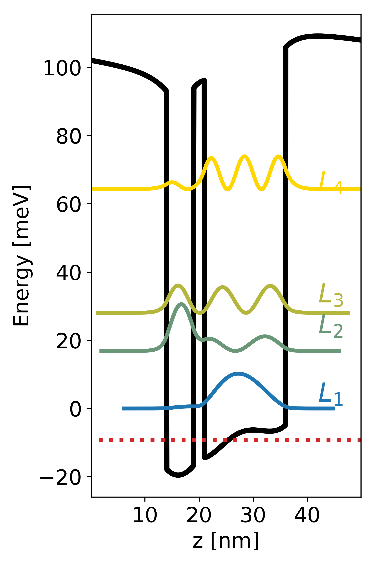

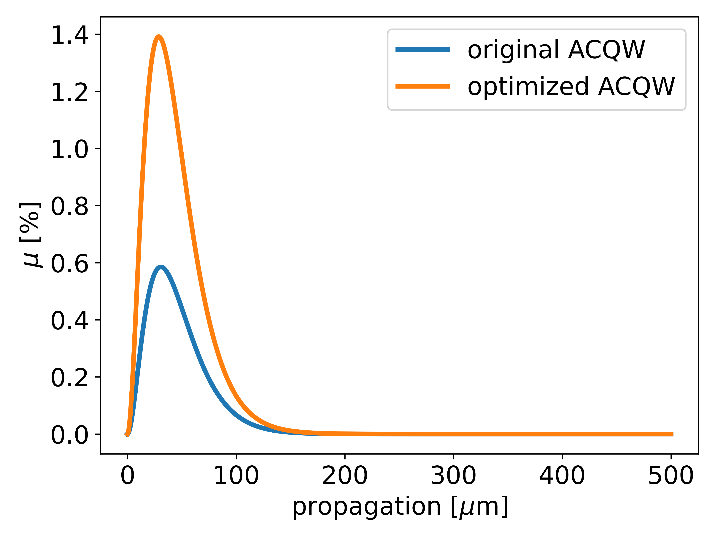


Figure S2: original (a) and optimized (b) ACQW structures for a doping concentration of 4x10^17^ cm^-3^.

c) comparison of their SHG efficiencies as a function of propagation length.

We point out that in principle it is possible to design a specific doping profile within the ACQW structure to minimize the variation in the intersubband spacing among the levels induced by a change in the doping concentration. As the placement of n-type dopant in Ge-rich alloys during epitaxy is notoriously difficult to control precisely, we preferred to avoid such theoretical speculations, instead assuming a homogeneous doping in both the QWs and the tunneling barrier.

From the calculations reported so far it seems that an increase in doping would lead to an increase in peak SHG efficiency. However, such calculations are based on the underlying and ultimately false assumption that the FWHM of the transition is independent of doping density. Due to the lack of systematic experimental data, it was not possible to derive a quantitative relation between FWHM and doping concentration. Qualitatively speaking, the FWHM increases with the doping [S1], resulting in an underestimation of the SHG efficiency below 1x10^17^ cm^-3^ and an overestimation above. Indeed, the main reason we specifically picked 1x10^17^ cm^-3^ as the doping density in the main text is the available experimental evidence suggesting that the chosen value of 6.5 meV for the FWHM represents a realistic, if not conservative, estimate [S2].

Finally, for the sake of speculation, we will briefly discuss the effect of doping on the modal effective refractive index. We start by pointing out how the real part of χ^1^ appears in equations (1) e (2) of the main text, which yield the relative permittivity of the ACQW region. In particular an higher χ^1^ leads to an higher permittivity. Since for TM modes it holds that Re{χ^1^(ω/2)} > Re{χ^1^(ω)}, we predicted the existence of a specific WG thickness where the impact on the effective mode refractive index brought about by the weaker confinement of TM_1_@ω/2 with respect to TM_1_@ω would be precisely counterbalanced by this permittivity difference. For a doping density of 1x10^17^ cm^-3^ we calculated such WG thickness to be 32.5 μm.

We mentioned how increasing the doping concentration boosts the magnitude of χ^1^ in both its real and imaginary parts, therefore increasing the permittivity. It follows that the aforementioned permittivity difference is likewise augmented, shifting the perfect phase mismatch condition towards thinner WGs. In Figure S3a we report the calculated effective refractive indexes seen by modes TE_1_@ω/2, TM_1_@ω/2 and TM_1_@ω as a function of WG thickness in a slab waveguide comprising ACQWs such as the one shown Figure S2b. Figure S3b displays the respective dephase lengths.


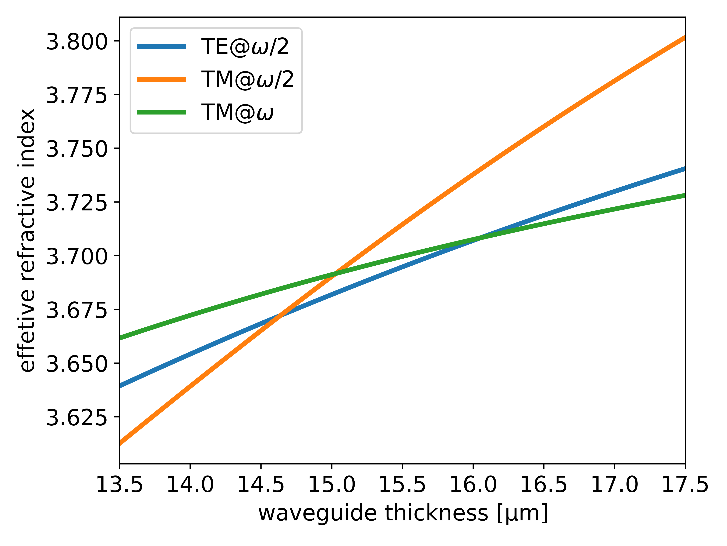

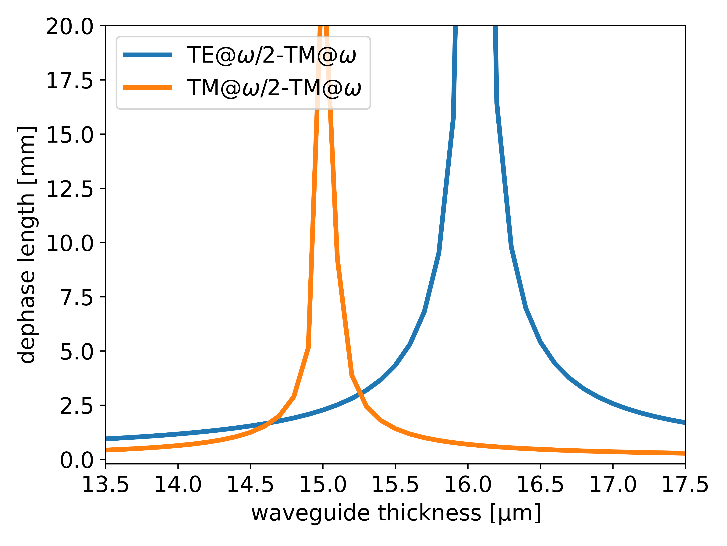


Figure S3: a) refractive indexes of modes TE_1_@ω/2, TM_1_@ω/2 and TM_1_@ω as a function of WG thickness. The ACQW included in the active region of the WG is the one shown in Figure S2b.

The perfect phase match is now achieved at a thickness of approximately 15.1 μm, leading to a corresponding divergence in dephase length. Interestingly, TE_1_@ω/2 also reaches a perfect phase match condition with TM_1_@ω at approximately 16.2 μm.

We conclude pointing out that these considerations regarding phase matching do not impact in any way the SHG efficiency. In fact, as a consequence of the large doping concentration, we are in regime where the efficiency is severely limited by absorption and phase mismatch does not play any relevant role.

**Considerations on temperature**

A variation in temperature affects the subband levels’ carrier populations. Specifically, as ISBTs at THz frequencies feature energies comparable with room temperature’s k_b_T, a strong increase in the first excited subband carrier population is expected as temperature rises, leading to a quenching of the χ^2^.

This is confirmed by the calculation of μ reported in Figure S4, performed at temperatures ranging between 25 and 300 K with a WG thickness of 15 μm, a pump frequency of 3.55 THz, a doping density of 10^17^ cm^-3^ and assuming a temperature invariant FWHM in line with Ref.[S1]. We find that the maximum SHG efficiency is reduced by a factor of ~20 when temperature is increased from 77 to 300K, and by a factor of ~5 at 200K. An analogous behavior with temperature is expected for any combination of the other device parameters.


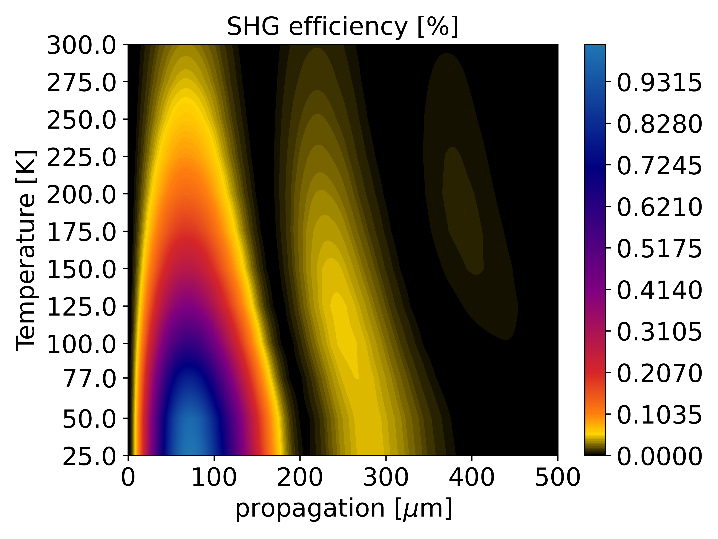


Figure S4: SHG efficiency μ as a function of temperature.

[S1] M. Virgilio, D. Sabbagh, M.Ortolani et al., “Physical mechanisms of intersubband-absorption linewidth broadening in s-Ge/SiGe quantum wells,” *Phys. Rev. B,* vol. 90, no. 15, pp. 155420, 2014, https://doi.org/10.1103/PhysRevB.90.155420.

[S2] C. Ciano, M. Virgilio, M. Montanari, et al., “Control of Electron-State Coupling in Asymmetric Ge/SiGe Quantum Wells,” *Phys. Rev. Appl.*, vol. 11, no. 1, pp. 014003, 2019, https://doi.org/10.1103/PhysRevApplied.11.014003.
